# Case Report: Anti-MDA-5 dermatomyositis in a resource-limited setting

**DOI:** 10.12688/wellcomeopenres.18728.1

**Published:** 2023-01-05

**Authors:** Ujjwol Risal, Suravi Pandey, Raju Pangeni, Rakshya Pandey, Dharmagat Bhattarai, Sudeep Adhikari, Buddha Basnyat

**Affiliations:** 1 Hospital for Advanced Medicine and Surgery, Kathmandu, Nepal; 2 Om Hospital and Research Centre, Kathmandu, Nepal; 3 Pyuthan Hospital, Pyuthan, Nepal; 4 Patan Hospital, Lalitpur, Nepal

**Keywords:** Anti-MDA-5, Dermatomyositis, Resource-limited setting

## Abstract

Anti-Melanoma Differentiation-Associated gene 5 (Anti-MDA-5) dermatomyositis is a rare subtype of inflammatory myopathy characterized by unique skin lesions, rapidly progressive interstitial lung disease, and skeletal muscle inflammation. It has a high mortality rate in the absence of early treatment. However, diagnosis of this entity is challenging in a country like Nepal because of various constraints such as lack of expert rheumatologists and resource limitations. Here we describe a case of one patient who had presented to us with generalized weakness, cough and shortness of breath who was finally diagnosed as anti-MDA-5 dermatomyositis. He responded to combination of immunosuppressives and is currently doing well. This case highlights the diagnostic and therapeutic challenges in managing such cases in a resource-limited setting.

## Introduction

Anti-Melanoma Differentiation-Associated gene 5 -Dermatomyositis (MDA5, DM) is a recently recognised subtype of myositis characterised by rapidly progressive interstitial lung disease (RP-ILD) and unique cutaneous features ^1^. However there are many challenges in the diagnosis and management of this disease especially in a resource-limited setting. Here we describe a case of ani-MDA-5 DM in a farmer from a hilly remote district of Nepal.

## Case summary

A 50-year-old male from a remote village in Western Nepal presented to us with difficulty standing from sitting position and performing overhead activities for six months’ duration. According to the patient, he initially had difficulty in standing from squatting position which progressed to involve his arms within two weeks. With delay in seeking medical help his limb weakness quickly progressed leaving him bed-bound within a period of one month. He had no difficulty holding objects in his hands nor did he complain of slippage of sandals which would indicate weakness of foot muscles. There was no difficulty in chewing or swallowing, double vision, blurring of vision, regurgitation of food, slurring of speech, or deviation of face. He did not complain of fluctuating weakness. There were no symptoms of sensory loss, or paraesthesia. There was no history of cold or heat intolerance, tremors, or abnormal sweating. His bladder and bowel functions were normal.

He also complained of bilateral knee pain which was more severe in the morning and was associated with swelling and warmth but no redness. There was early morning stiffness of knee joints which lasted for around one hour. No other joints were involved. He had severe fatigue since the onset of illness with significant weight loss. There was complaint of excessive hair loss. He denied presence of rash or symptoms suggestive of Raynaud’s phenomenon.

Apart from the muscle weakness, he gave history of a dry cough for six months which was initially occasional but had progressed to become severe for the past couple of months. There was progressive shortness of breath on exertion since his cough began. He denied any history of bloody sputum or red coloured urine. He was being seen by a physician in his town and his symptoms were being managed as gout with low dose steroid, allopurinol, and colchicine. However, his symptoms kept progressing despite several days of hospital admission.

**Figure 1.**
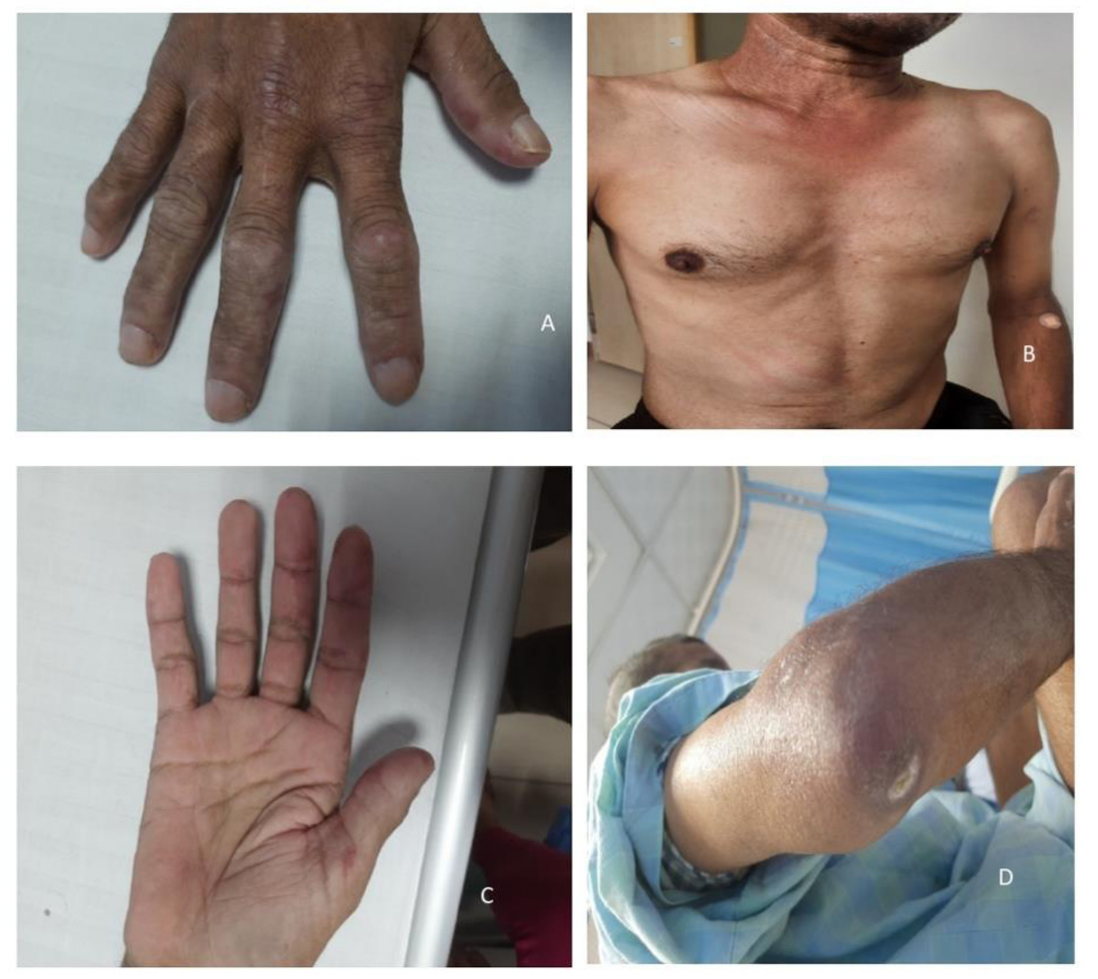
**A**. Gottron’s papules involving the dorsal aspect of proximal interphalangeal (PIP) joints **B**. ‘V’ sign: Erythematous patch involving the ‘V’ of neck **C**. Palmar papules: Erythematous macules involving bases of thumb of right-hand **D**. Calcinosis involving right elbow.

On examination, the patient was emaciated with stable vitals and oxygen saturation of 96% on room air. He had diffuse alopecia. Hand examination showed multiple erythematous macules on the palmar aspect and multiple hypo-pigmented macules on the dorsal aspect of proximal-inter-phalangeal (PIP) joints ( Figure 1). His nails showed fraying of cuticles. Examination of the elbows showed denuded epithelium with scab formation on one side and chalky white deposits on the other ( Figure 1). There was an erythematous rash involving the anterior aspect of neck and upper chest ( Figure 1). He had generalized muscle atrophy. Power assessment showed weakness of neck flexors and extensors (2/5), shoulders (4-/5 in all ranges of motion), hips (extension 3/5, flexion 4-/5 with normal adduction and abduction), and knees (flexion 3/5 and extension 5/5). His sensory, cranial nerves, and deep tendon reflexes were normal. His respiratory system examination revealed bilateral infrascapular Velcro crackles. Examination of the knees showed features of inflammation with decreased range of motion. Passive movement of his shoulders was painful and limited. Other systemic examination was normal. With the presence of features of symmetrical myopathy with skin changes, respiratory system involvement and arthritis, a provisional diagnosis of dermatomyositis was made.

**Figure 2.**
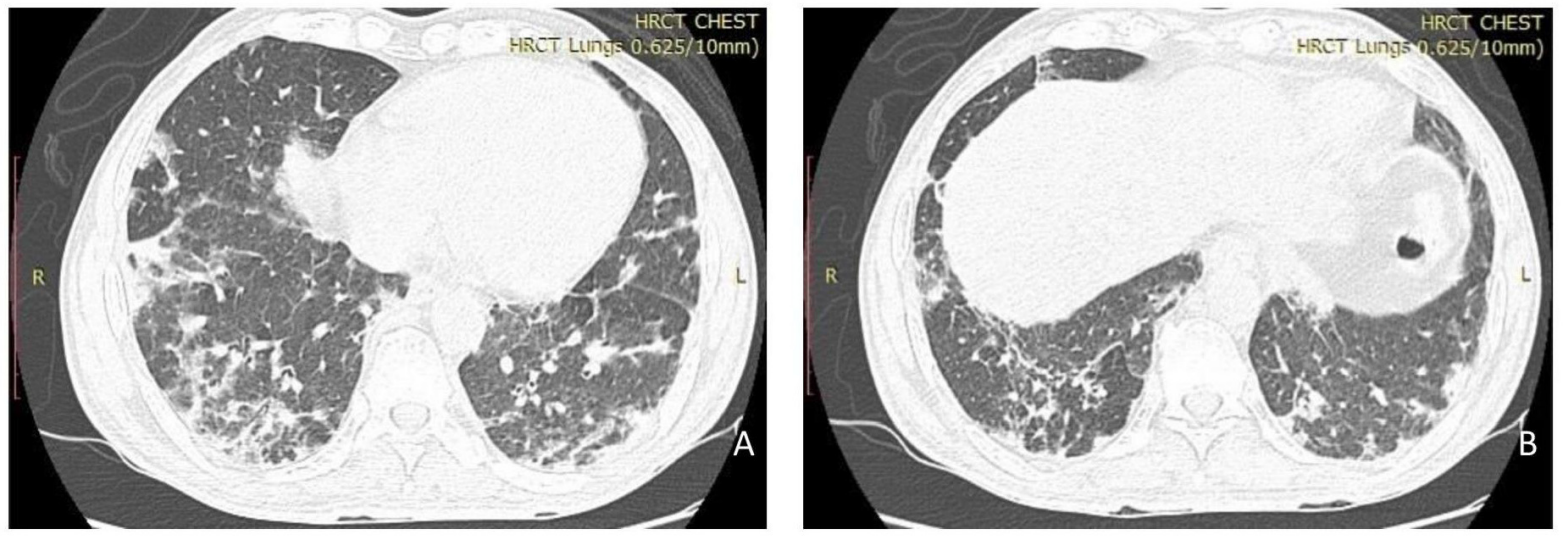
HRCT lungs- Bilateral peripheral lower lobe predominant reticulation with patchy consolidation and ground glass changes.

Muscle enzymes, anti-nuclear antibody (ANA), anti-ENA (Extractable nuclear antigen), and muscle specific antibody (MSA) were ordered. High resolution computer tomography (HRCT) of the chest showed features of non-specific interstitial pneumonia (NSIP) pattern of interstitial lung disease (ILD) [ Figure 2]. Muscle enzymes including creatine phosphokinase (CPK), aspartate and alanine transaminases (AST and ALT), and lactate dehydrogenase (LDH) were all raised ( Table 1). An MSA profile report was awaited. With the working diagnosis of dermatomyositis with ILD, the patient was immediately started on prednisolone at 1mg/kg and azathioprine 50 mg per day along with active and passive physiotherapy of his muscles. With this treatment, his knee pain and cough improved. He was discharged after a couple of days with the final diagnosis of DM with ILD with a follow up visit arranged within one week with the MSA panel result.

**Table 1.** Laboratory parameters of the patient during admission, at the time of second discharge (two weeks from initial presentation), and at follow-up four months after admission.

	At admission (normal range in parenthesis)	At the time of second discharge (two weeks)	At follow-up (four months)
Hemoglobin (gm/dl)	11.8 (13.8–17.2)	12.2	13.2
White cells (/mm ^3^)	4950 (4500–11000)	7510	9470
Platelet (/mm ^3^)	227000 (150000–450000)	341000	346000
C-Reactive protein (mg/L)	29.9 (0–6 )	1.12	11.03
Creatine phosphokinase- Total (U/L)	228.3 (55–190)	30.1	46
Aspartate transaminase (U/L)	130 (8–48)	43	29
Alanine transaminase (U/L)	99 (7–55)	32	46
Sodium (mmol/L)	133 (135–145)	137	140
Potassium (mmol/L)	3.6 (3.5–5.5)	3.5	3.8
Lactate edhydrogenase (U/L)	698 (105–333)	242	
Erythrocyte sedimentation rate (mm/hr)	40 (<15)		35
Ferritin (ng/ml)	1270 (30–400)		

**Figure 3.**
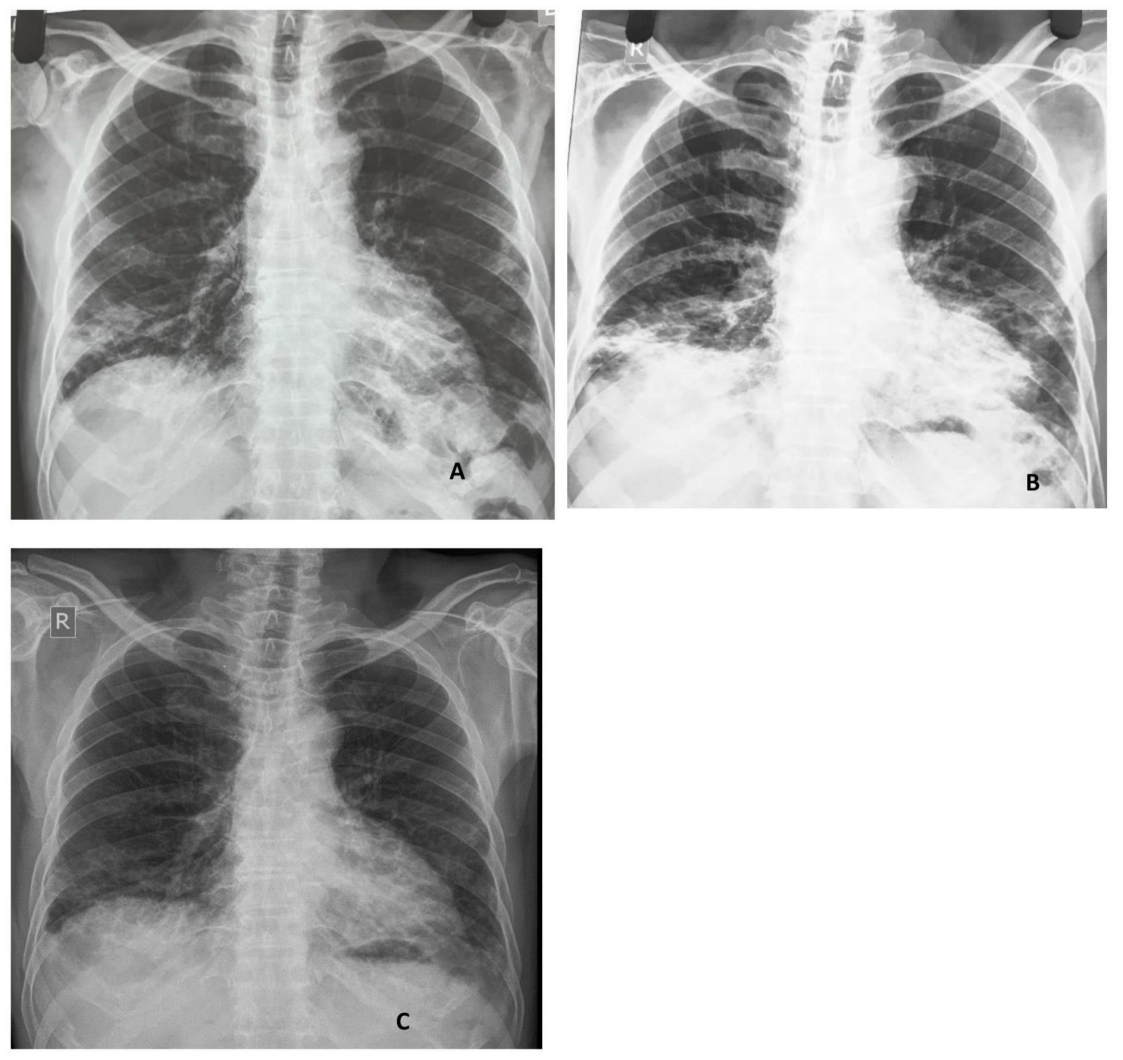
**A** and **B**: Chest x-rays: Initial and two weeks following treatment, findings show bilateral lower zone (right>left) peripheral airspace changes. **C**. Two months after initiation of immunosuppression, improvement in bilateral airspaces changes.

However, after two days of discharge, the patient presented to out-patient clinic with increasing shortness of breath and cough. On examination, his oxygen saturation was 93% on room air and was tachypnoeic. His chest X-ray showed increased infiltrates in bilateral lung fields ( Figure 3). He was admitted and given pulse intravenous methylprednisolone one gram/day for three days. Broad-spectrum antibiotics were also started to cover any possible infection. Meanwhile, MSA profile came back which showed strongly positive anti MDA-5 antibodies.

 A final diagnosis of anti-MDA-5 DM was made. He was scheduled for further immunosuppression with pulse cyclophosphamide, tacrolimus, and mycophenolate mofetil. Since he was from a remote village in Western Nepal, starting him on a calcineurin inhibitor was not feasible as frequent laboratory monitoring would not be possible. The option of monthly pulse of intravenous (IV) cyclophosphamide and mycophenolate mofetil was discussed with the patient which he accepted. Azathioprine was stopped. The first cycle of IV cyclophosphamide was started together with mycophenolate mofetil and high dose oral prednisolone. His oxygen requirement started decreasing and his muscle power started improving in a couple of days. At the time of discharge, he was off oxygen and was able to mobilise on his own without any walking aid. His laboratory parameters including muscle enzymes also improved markedly ( Table 1). At the time of writing this manuscript, our patient had already completed three cycles of pulse cyclophosphamide and had no clinical or laboratory issues.

On follow-up during his second cycle of IV cyclophosphamide, patient remained well with full power in his limbs with no muscle weakness, cough, or shortness of breath. We plan to complete six cycles of cyclophosphamide with daily mycophenolate mofetil and slow tapering of prednisolone together with close follow-up.

## Discussion

Anti-Melanoma Differentiation-Associated gene 5 (MDA-5) Dermatomyositis (MDA5, DM) is a rare systemic autoimmune disease initially described in Japanese patients with clinically amyopathic dermatomyositis (CADM) and rapidly progressive interstitial lung disease (ILD) ^2^. MDA5, DM is characterised by a typical DM rash, polyarthralgia, and ILD. The clinical signs of myositis are usually absent in these cases ^3^. MDA-5 was first identified in 2002 which is now considered a key protein in mediating an antiviralresponse ^4^. The frequency of antiMDA-5 DM can range from 7-10%in European population to as much as 25% in the Japanese population amongst all adult DM cases ^5, 6^. Because of the widespread availability of various muscle specific antibodies, this entity is being increasingly recognised all over the world. Moreover, three phenotypes of MDA5 DM have been identified which have certain distinct characteristics. Phenotype 1 which consists of 20% of cases is usually seen in women and has arthralgia/arthritis as the main feature. Phenotype 2 is more common in male and consists of 50% of cases which is characterised by proximal muscle weakness and skin vasculopathies. Phenotype 3 is seen in 30% of cases and is more common in female and is associated with mechanic’s hands ^2^. Since our patient had proximal muscle weakness along with skin changes, he most likely belonged to phenotype 2.

The diagnosis of Anti-MDA-5 DM is a huge challenge in a country like Nepal where rheumatology is a budding subspeciality of internal medicine and where there are only few rheumatologists who are mostly practising in the capital of the country. Our patient was a farmer who belonged to a rural village in the Western part of Nepal and had limited access to health services. Even though he did visit the nearest town for his ailment, his diagnosis was delayed because of a lack of proper training and awareness among clinicians who were managing this case as gout. Moreover, he was advised not to take any protein in his diet saying it would aggravate his symptoms since it is common practice among many doctors to restrict protein in diet in any case of arthritis or arthralgia. Diagnostic delay and severe protein restriction on top of the progressive disease itself were probably responsible for his rapid deterioration.

Despite recognizing the disease on the first day of presentation, we faced many challenges ourselves, the most important one being the lack of availability of myositis specific antibody (MSA) testing in Nepal. The test had to be sent to India which was time-consuming and expensive. The second challenge was the patient’s poor financial condition and lack of disease awareness and insight. The third challenge we faced was the lack of clear-cut treatment guidelines for the management of anti-MDA5 DM. The disease itself has a poor prognosis with a mortality rate of as much as 80% ^7^. Moreover, our patient had high ferritin level and lung involvement which are both considered to be poor prognostic markers ^8^. There are no guidelines for the management of anti-MDA5-DM. However, a combination of immunosuppressives with mycophenolate mofetil (MMF), pulse cyclophosphamide, and a calcineurin inhibitor along with high dose steroids have been widely used with success ^8^. Here also we had to choose the most feasible form of treatment which fortunately led to good clinical outcome in our patient.

The management of this patient highlights the difficulties and uncertainties faced by clinicians in managing various rheumatological disorders in a resource limited country like Nepal.

## Conclusion

Anti-MDA5-DM is a rare but rapidly progressive disease whose diagnosis and treatment pose a huge challenge especially in a resource-limited setting like Nepal. Awareness of the disease among clinicians, easy availability of diagnostic tests, and treatment are some of the ways that could help in tackling these challenges.

## Informed Consent

Written informed consent for the publication of their clinical details and images was taken from patient before submission.

## Data Availability

All data underlying the results are available as part of the article and no additional source data are required.
