# Supplementary material for: Case Report: Anti-MDA-5 dermatomyositis in a resource-limited setting
Source: Wellcome Open Res. 2023 Jan 5;8:5. [Version 1] doi: 10.12688/wellcomeopenres.18728.1 (PMC10220425; doi:10.12688/wellcomeopenres.18728.1)
Supplement: Peer review history

# Peer review: Reviewer response for version 1

**DOI:** 10.21956/wellcomeopenres.20766.r57292

**Published:** 2023-05-26

**Authors:** Liza Rajasekhar

**Affiliations:** 1 Nizam's Institute of Medical Sciences, Hyderabad, Telangana, India

This report which describes the evolution of anti-MDA5 disease in a patient from Nepal will be of great local relevance to the general medical practitioners, postgraduates of the country. The treatment with a combination of cyclophosphamide and Mycophenolate however goes beyond the recommendations of using a single immunosuppressive. It may have been the best judgement of the clinician's.

 The merit of the case lies in making a definitive diagnosis in line with the modern methods of diagnosis available.

Are enough details provided of any physical examination and diagnostic tests, treatment given and outcomes?

Yes

Is the case presented with sufficient detail to be useful for other practitioners?

Yes

Is sufficient discussion included of the importance of the findings and their relevance to future understanding of disease processes, diagnosis or treatment?

Yes

Is the background of the case’s history and progression described in sufficient detail?

Yes

Reviewer Expertise:

Lupus Myositis

I confirm that I have read this submission and believe that I have an appropriate level of expertise to confirm that it is of an acceptable scientific standard.

# Peer review: Reviewer response for version 1

**DOI:** 10.21956/wellcomeopenres.20766.r57294

**Published:** 2023-05-25

**Authors:** David F. Fiorentino

**Affiliations:** 1 Stanford University, Stanford, California, USA

This is a very well written and interesting case presented of anti-MDA5 disease managed in a resource-limited setting. The authors make several good points about peculiar challenges associated with managing this disease in this environment. I have just a couple of suggestions:

- Did the patient have any dysphonia?
- On physical exam, please note that the alopecia was non-scarring?
- On the chest CT - any features of organizing pneumonia (which is a common finding in this subgroup) or fibrosis?
- The description of 3 phenotypes probably should be omitted. This was one study, in Europe, and really of unclear significance at this time. Alternatively, please put this study in context (it was a single study - it is not the final word) and the authors suggested 3 phenotypes. Probably wouldn’t put your patient into a phenotype as this suggests that this classification is universal and validated.
- The authors state that in anti-MDA5 disease, clinical signs of myositis are usually absent. Please consider rewording this noting that, although initially described this way in the Japanese literature, it is clear that many of these cases can have significant muscle involvement, and this should not dissuade the clinician from making this diagnosis.
- Also, it is worth noting that ILD is not always associated with MDA5, and, furthermore, that, even when present, there is a significant group of patients (especially in non-Asians) that experience more subacute ILD (not RP ILD) and do very well with therapy. Would qualify that the disease has a poor prognosis **in cases where RP ILD is present** only.
- Interesting that, even in a resource limited setting, the clinicians were able to obtain results of anti-MDA5 testing, and quite rapidly. I would say availability of reliable antibody testing would be another challenge in certain settings - especially given that the reliability of these tests varies greatly by methodology. A high ferritin test can support a diagnosis of anti-MDA5 if antibody tests are not available.

Are enough details provided of any physical examination and diagnostic tests, treatment given and outcomes?

Yes

Is the case presented with sufficient detail to be useful for other practitioners?

Yes

Is sufficient discussion included of the importance of the findings and their relevance to future understanding of disease processes, diagnosis or treatment?

Yes

Is the background of the case’s history and progression described in sufficient detail?

Yes

Reviewer Expertise:

autoantibodies and dermatomyositis

I confirm that I have read this submission and believe that I have an appropriate level of expertise to confirm that it is of an acceptable scientific standard, however I have significant reservations, as outlined above.

# Peer review: Reviewer response for version 1

**DOI:** 10.21956/wellcomeopenres.20766.r56302

**Published:** 2023-05-09

**Authors:** Pin-Kuei Fu

**Affiliations:** 1 Department of Medical Research, Taichung Veterans General Hospital, Taichung, Taiwan

This is a compelling case of anti-MDA5-positive dermatomyositis diagnosed in Nepal, a resource-limited country. The patient exhibited phenotype 2, presenting with proximal muscle weakness and skin vasculopathies. The case report is well-written and well-organized. It is suggested that in the first part of the discussion, additional information regarding the risk factors or biomarkers associated with MDA5-DM-ILD may be helpful for readers to recognize this potentially fatal disease.

Are enough details provided of any physical examination and diagnostic tests, treatment given and outcomes?

Yes

Is the case presented with sufficient detail to be useful for other practitioners?

Yes

Is sufficient discussion included of the importance of the findings and their relevance to future understanding of disease processes, diagnosis or treatment?

Yes

Is the background of the case’s history and progression described in sufficient detail?

Yes

Reviewer Expertise:

Interstitial lung disease, Biomarkers

I confirm that I have read this submission and believe that I have an appropriate level of expertise to confirm that it is of an acceptable scientific standard.
